# Expansion of the Preimmune Antibody Repertoire by Junctional Diversity in *Bos taurus*

**DOI:** 10.1371/journal.pone.0099808

**Published:** 2014-06-13

**Authors:** Jenni Liljavirta, Mikael Niku, Tiina Pessa-Morikawa, Anna Ekman, Antti Iivanainen

**Affiliations:** Department of Veterinary Biosciences, University of Helsinki, Helsinki, Finland; Chang Gung University, Taiwan

## Abstract

Cattle have a limited range of immunoglobulin genes which are further diversified by antigen independent somatic hypermutation in fetuses. Junctional diversity generated during somatic recombination contributes to antibody diversity but its relative significance has not been comprehensively studied. We have investigated the importance of terminal deoxynucleotidyl transferase (TdT) -mediated junctional diversity to the bovine immunoglobulin repertoire. We also searched for new bovine heavy chain diversity (*IGHD*) genes as the information of the germline sequences is essential to define the junctional boundaries between gene segments. New heavy chain variable genes (*IGHV*) were explored to address the gene usage in the fetal recombinations. Our bioinformatics search revealed five new *IGHD* genes, which included the longest *IGHD* reported so far, 154 bp. By genomic sequencing we found 26 new *IGHV* sequences that represent potentially new *IGHV* genes or allelic variants. Sequence analysis of immunoglobulin heavy chain cDNA libraries of fetal bone marrow, ileum and spleen showed 0 to 36 nontemplated N-nucleotide additions between variable, diversity and joining genes. A maximum of 8 N nucleotides were also identified in the light chains. The junctional base profile was biased towards A and T nucleotide additions (64% in heavy chain VD, 52% in heavy chain DJ and 61% in light chain VJ junctions) in contrast to the high G/C content which is usually observed in mice. Sequence analysis also revealed extensive exonuclease activity, providing additional diversity. B-lymphocyte specific TdT expression was detected in bovine fetal bone marrow by reverse transcription-qPCR and immunofluorescence. These results suggest that TdT-mediated junctional diversity and exonuclease activity contribute significantly to the size of the cattle preimmune antibody repertoire already in the fetal period.

## Introduction

Somatic recombination generates a large immunoglobulin repertoire by the assembly of variable (V), diversity (D) and joining (J) gene segments coding for heavy chains and V and J segments coding for light chains [1]. In cattle and several other domestic animals the germline population of V, D and J segments is too small to provide sufficient immunoglobulin diversity. These species use additional mechanisms in order to expand the preimmune repertoire, which is the repertoire in use before exposure to environmental antigens [2]. Long immunoglobulin heavy chain D genes are characteristic of bovine immunoglobulins as they contribute to the exceptionally long third complementarity determining regions of the heavy chains (CDR3H) [3]–[5]. We have previously shown that somatic hypermutation (SHM) diversifies the immunoglobulin repertoire by introducing mutations especially in the CDR3H region, already at the fetal period, before the exposure to external antigens [6]. In addition to SHM, terminal deoxynucleotidyl transferase (TdT) mediated junctional diversity has been reported in cattle but its significance to the preimmune repertoire has not been thoroughly investigated [7].

TdT adds nontemplated (N) nucleotides to the single-strand DNA ends, in connection with V(D)J recombination which is guided by recombination signal sequences (RSSs). These conserved sequences flank each V, D and J segment. [1]. The recombination process requires multiple enzymes such as polymerases, nucleases and ligases. A complex encoded by recombination-activating genes (*RAG1* and *RAG2*) plays a crucial role in bringing the two RSSs together and cleaving the double stranded DNA. As a result, the cleaved free end forms a DNA hairpin which is then opened by the Artemis:DNA-dependent protein kinase (DNA-PK) nuclease complex at a random site. Sometimes the cleavage generates palindromic (P) nucleotides [8]. Whenever TdT is present and active in the cell, it increases the variability of the junctions by adding N nucleotides to the available 3′-OH ends adjacent to the P nucleotides. Also excision of nucleotides by largely uncharacterized exonucleases occurs [9]. As the N- and P-nucleotide additions are largely random they often result in nonproductive rearrangements [10], [11]. In mice, the length of productive N additions is 2–5 bp *in vivo*. *In vitro* experiments have shown that TdT is capable of catalyzing even longer than 1 kb nucleotide additions [12] with a bias towards dGMP residues [13]. In addition to rearranged immunoglobulin genes, N additions also take place in genes encoding T-cell receptors [14].

TdT belongs to the PolX family of DNA polymerases with Polβ, Polλ and Polµ in eukaryotes [15]. It is considered the only canonical template independent DNA polymerase, although Polµ has also been reported to have template independent functions [16]. In mammals, alternative splicing generates two or three TdT isoforms among which functional differences have been observed. In mouse two isoforms, mTdTS and mTdTL have been identified [17]. All of the murine isoforms are expressed after birth and N additions are usually found only in rearranged IGH genes. The function of mTdTL still remains unclear. It is suggested that rather than adding nucleotides it may function as an exonuclease, trimming the coding ends of V, D and J segments [18], [19]. Human and cattle have three isoforms: TdTS, TdTL1 and TdTL2 [20], [21]. In humans, both of the long isoforms possess 3′→5′ exonuclease activity. Human TdTS, on the contrary, may carry out nucleotide elongation during V(D)J recombination. The human TdTs are expressed already in fetal life in T- and B-cell progenitors in thymus and bone marrow [21].

In this study, we first complemented the current *IGH* gene repertoire by searching new immunoglobulin variable (*IGHV*) and diversity (*IGHD*) genes. Accurate reference germline sequences were a prerequisite for analysing the junctional boundaries in fetal recombinations. Junctional diversity was then analysed from fetal cDNA libraries of both heavy and light chains. Furthermore, the expression of TdT and its splice variants was investigated by reverse transcription (RT) qPCR and triple-colour immunofluorescence in several tissues. We focused on fetal recombinations as in cattle *de novo* B lymphopoiesis takes place in fetal bone marrow and lymph nodes, as indicated by expression of pre-B cell markers [22], [23]. Our results indicate significant TdT-induced junctional diversity in bovine immunoglobulins and suggest a novel diversification mechanism which involves extensive trimming of *IGHD* genes.

## Materials and Methods

### Ethical statement

Fetal and adult tissue samples were collected from a local abattoir, where cattle are slaughtered on a daily basis and used for human consumption. We took our samples from slaughtered, healthy animals. No experimental procedures were done on living animals and they were not euthanized for this study. Therefore, no ethical permit was required.

### Cloning and sequencing of bovine germline IGHV genes

Germline *IGHV* genes were cloned and sequenced as previously described [6]. Skeletal muscle genomic DNA was extracted from three fetuses, (aged 182, 240 and 270 gestation days (gd)) and a 51-days-old calf. The reads of an *IGHV* were considered reliable if the same sequence was identified at least twice. The new *IGHV* sequences are presented in Table S1.

### Extraction of bovine IGHD sequences from genomic sequencing data

Available bovine genomic sequencing data (including the NCBI Unfinished high throughput genomic sequences and the NCBI trace archive) was explored for previously unidentified IGHD genes using the fuzznuc motif search [24] for consensus D-RSS sequences. The PROSITE search motives used were GGTTTTTGT N(11,13)CACNGTGN(6,160)CACNGTGN(11,13)ACAAAAACC with up to 4 mismatches and GG[TA]TTN[ATG][GA][ATG]N(12)N[AG][TC]NGT[GC] N(30,180) CAC[ACT][AG][TC][GA] N(12)NC[AC][ACG]AAA[ACG][CT] with up to 2 mismatches. The known and newly identified *IGHD* genes are presented in the Table S2.

### Preparation of fetal IGH, IGL and IGK cDNA libraries

For the heavy chain library, samples of ileum, spleen and bone marrow were collected from fetuses of 240 and 270 gd. First-strand cDNA was synthesised using SuperScript III First-Strand Synthesis SuperMix according to manufacturer's instructions. First-strand cDNA and the IGH cDNA library was prepared as described in [6].

For the light chain libraries, total RNA was purified from ileum and bone marrow of 270 gd old fetus. The first-strand cDNA was primed using equal amounts of oligo (dT)_20_ and random hexamer primers and SuperScript III First-Strand Synthesis SuperMix was used for cDNA synthesis. Furthermore, the variable and joining segments were amplified with PCR using Phusion MasterMix (Fermentas). The PCR reaction contained 1 x Phusion MasterMix, 0.5 µM forward and reverse primers (IgLg1fwd and IgLg1 rev for lambda light chains and IgKg2 fwd and IgKg2 rev for kappa light chains, Table 1) and 0.5 µl of the cDNA template (ileum or bone marrow). Two-step PCR protocol cycling conditions consisted of an initial denaturation of 98°C for 30 s, followed by 29 cycles of 98°C for 10 s, 72°C for 15 s, and a final extension of 72°C for 7 min. PCR products were then electrophoresed and purified. Approximately 20 ng of the purified PCR product was ligated into the pCR Blunt II-TOPO Vector and transformed into TOP10 *E. coli* (Life technologies). The TOP10 *E. coli* were grown overnight at 37°C on LB-kanamycin (50 µg/ml) plates. A total number of 48 single colonies were picked up, purified and sequenced by GATC Biotech AG (Konstanz, Germany).

**Table 1:** PCR primers and probes.

Primer	Sequence 5′-3′
**TdT-FW**	GCTTCAGGTACAGAACATA
**TdT-REV**	GTCTGTTCTCACAACAAG
**TdT probe**	FAM-ACT[+C]CT[+T]GA[+T]GT[+C]TCCTG-BHQ1*
**T7**	TAATACGACTCACTATAGGG
**T3**	ATTAACCCTCACTAAAGGGA
**TdT_tr1_1925_FW**	AGACCAAGTGCACATATG
**TdT_tr1_1925_PR**	FAM-ATTTCTTCTTCACTTTCCGCTTTGAGA-BHQ1
**TdT_tr1_1925_RV**	GGTTCAATGTAGTCCAATC
**TdT_tr2_131_FW**	TGGTCAGGTTTTGGATTTC
**TdT_tr2_131_PR**	HEX-CAGAAATGCCCACACAGCCTC-BHQ1
**TdT_tr2_131_RV**	CCTGTCATGGTGACAAAG
**18S fwd**	TGGTTGCAAAGCTGAAACTTAAAG
**18S probe**	HEX-CCTGGTGGTGCCCTTCCGTCA-BHQ1
**18S rev**	AGTCAAATTAAGCCGCAGGC
**IgLg1fwd**	GGCCCAGGCTGTGCTGACTC
**IgLg1 rev**	TGATGGTGCTGCCGTCTGCC
**IgKg2 fwd**	TGTGCTGACCCAGACTCCCCT
**IgKg2 rev**	ACAGTTCCGGTCTTCAGCTGCTC
**IgH fwd1**	TTGTGCTSTCAGCCCCCAGA
**IgH rev1**	CGCAGGACACCAGGGGGAAG
**IGHV fwd3**	GGACAACTCCAAGAGCCAAG
**IGHJrev2-FAM**	TGAGGAGACGGTGACCMKGAG

*Nucleotides in square brackets refer to locked nucleic acids.

### Spectratyping

Total RNA was extracted as in the previous sections. The first-strand cDNA was reverse transcribed using RevertAid Premium Reverse Transcriptase (Fermentas) and primed with equal amounts of oligo (dT)_20_ and random hexamer primers. First-strand cDNA was used as a template for the nested PCR. For the first round primers IgH fwd1 and IgH rev1 were used, amplifying the region from leader1 exon to the CH1 region. This PCR product was used as a template for the second round PCR. Here, primers covering the CDR3H region were used (IGHV fwd3 and IGHJ rev2-FAM, Table 1). Capillary electrophoresis was run at the Sequencing unit of the Institute of Biotechnology (University of Helsinki). The raw data were analysed in PeakScanner (ABI). Data was filtered and combined from 24 samples (four fetuses, six tissues: bone marrow, ileum, liver, lymph node, spleen, and thymus), and the signal density function with Gaussian smoothing kernel was computed in R [25].

### Sequence analysis of V(D)J junctions

The sequence data from cDNA libraries were analysed with Geneious Pro software version 6.0 (Biomatters, New Zealand), the EMBOSS package [24], MUSCLE version 3.7 [26], and R software [25]. Sequences were discarded when they did not cover the entire CDR3 region. Sequences were aligned with previously detected germline sequences [3], [27]–[30] (see also Tables S1, S2 and S3) using Smith-Waterman local alignment algorithm implemented in Biostrings R-package [31] and its heuristic approximation implemented in blastn [32]. The heavy chain cDNA sequences were aligned against custom bovine-specific *IGHV, IGHD and IGHJ* gene databases by the pairwiseAligment function in Biostrings using the following parameters: match = 1, mismatch = -1, gapOpening = -4, gapExtension = -5 (for variable and joining genes) or -0.3 (diversity genes), and type of aligment = local. The boundaries corresponding to the V, D and J segments derived from specific *IGHV*, *IGHD* and *IGHJ* germline sequences were determined from the coordinates of the best pairwise alignment with the query sequence. The boundaries for V and J segments were first determined and the subsequence between these coordinates was then used for querying the *IGHD* database (Table S4). The gap extension penalty was optimized to a low level (-0.3) to extend the alignments, since the *IGHD* sequences contain shared repetitive short motifs (Table S2). Boundaries for overlapping V, D and J segments were set at the middle of the overlap. The light chain sequences were analysed in a similar way except that blastn was used for pairwise alignments.

Recombination-associated exonuclease activity was determined for each end of the germline reference gene acting as the donor sequence (Table S5). For this, we counted the number of donor sequence nucleotides excluded from the recombined sequence. When the donor sequence end had not been modified, potential P nucleotides complementary to the donor sequence end were identified. The reverse complement of the donor sequence end was compared nucleotide by nucleotide with the recombined query gene sequence in the VD or DJ junction. The remaining nucleotides in the VD or DJ junction were classified as N nucleotides (Tables S4 to S7).

### Assessment of the expression of TdT splice variants by reverse transcription-qPCR (RT-qPCR)

Tissue samples from 6 fetal and 5 adult cattle were collected from a local abattoir, snap frozen in liquid nitrogen, and stored at −80°C. Total RNA was extracted from liver, ileum, spleen, lymph node, thymus and bone marrow using Eurozol (EuroClone, Italy) as described in [6].

Reverse transcription into cDNA was performed using 1 µg of total RNA with Revert-AID M-MuLV Reverse Transcriptase (Fermentas, Germany) or SuperScript III First-Strand Synthesis SuperMix (Life technologies, Germany). First-strand cDNA was primed with oligo(dT)_20_ (Oligomer, Finland) and synthesis was performed according to manufacturer's instructions.

**Figure 1:**
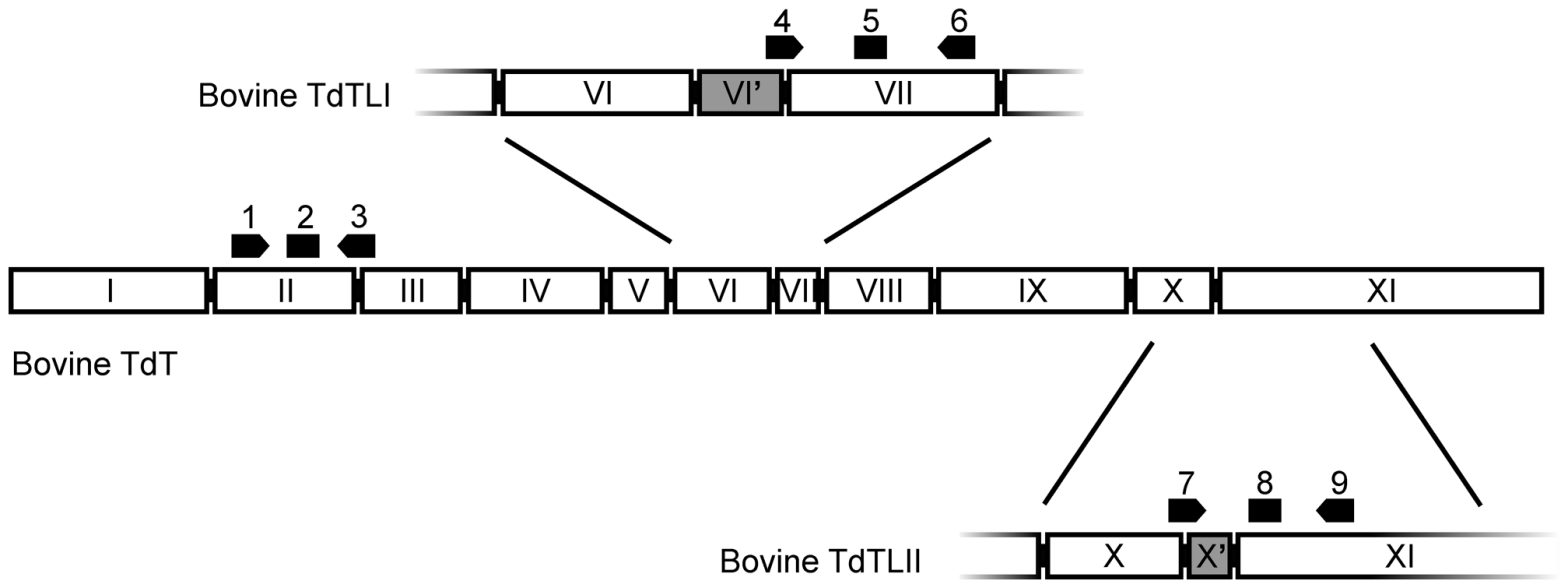
qPCR primers and probes for bovine TdT splice variants. Numbering of exons according to human TdT [68]. Extra exons in the splice variants (VI’ and X’) are numbered according to [20]. Black arrows indicate the primers and rectangles the probes for qPCR, as specified in Table 1. 1. TdT-FW, 2. TdT probe, 3. TdT-REV, 4. TdT_tr2_131_FW, 5. TdT_tr2_131_PR, 6. TdT_tr2_131_RV, 7. TdT_tr1_1925_FW, 8. TdT_tr1_1925_PR, 9. TdT_tr1_1925_RV.

Three sets of primers and probes were designed for the different splice variants (Figure 1 and Table 1). TdT-FW and TdT-REV recognize all three forms of the bovine TdT mRNA. TdT_tr_1_1925_FW and TdT_tr_1_1925_RV recognize the splice variant II and the TdT_tr2_131_FW and TdT_tr2_131_RV recognize the splice variant I. All primers and probes were designed by Sigma-Aldrich. Amplification was carried out using the Stratagene Mx3005P real-time PCR system (Agilent Technologies, USA). Cycling conditions were: 95°C for 10 min, followed by 40 cycles of 95°C for 15 s, 60°C for 30 s and 72°C for 30 s. Reactions were performed in duplicates.

TdT expression was quantified using RT-qPCR. From each tissue the threshold cycle for TdT was normalized with that of 18S RNA. In order to compare the relative changes in the splice variant I and II gene expression, the 2^−ΔΔCt^ method was used [33]. The 18S-normalized values were calibrated with the normalized value of the expression in the adult thymus. The range for relative expression was between 0 (no detectable expression) and 1 (the same level of expression as in the calibrator thymus). The statistical analysis was done in R with non-parametric Friedman two-way ANOVA followed by pair-wise comparison using Wilcoxon-Nemenyi-McDonald-Thompson test when assessing the all-over TdT mRNA expression. Kruskal-Wallis ANOVA followed by pair-wise comparison using Nemenyi-Damico-Wolfe-Dunn test was used with splice variant analysis [25], [34].

### Triple immunofluorescence and image analysis

Tissue sections were deparaffinized, subjected to heat-induced antigen retrieval in 10 mM Tris-HCl pH 9.5, 1 mM EDTA pH 8, and blocked with 1% goat serum. They were then incubated in a mixture of a rabbit polyclonal anti- bovine TdT antibody (Dako, Denmark) and the rat monoclonal anti-CD3 antibody CD3-12 (Santa Cruz Biotechnology, TX), washed, incubated in a mixture of a goat anti-rabbit Ig Alexa647 antibody (Dako) and a goat anti-rat Ig DyLight488 antibody (Jackson Immunoresearch, PA), washed again, incubated in the monoclonal mouse anti-human CD79α antibody HM57 (Dako), washed and finally incubated in a donkey anti-mouse Ig DyLight549 antibody preadsorbed against rat Igs (Jackson Immunoresearch). The sections were counterstained with DAPI, fixation autofluorescence was suppressed by incubation in 0.1% Sudan Black B in 70% ethanol, and the coverslips were mounted using Dako Immunofluorescence Mounting Medium.

The stained sections were photographed in the four fluorescence channels using a Zeiss AxioVision microscope. To assess the phenotypes of the TdT^+^ cells, photomicrographs of randomly selected TdT^+^ cells were observed in the CD3 and CD79α channels. CD79α^+^CD3^−^ cells were counted as B lymphocytes and all CD3^+^ cells as T lymphocytes. To calculate the proportions of TdT^+^ cells among all B lymphocytes, TdT^+^CD79α^+^CD3^−^ cells were first manually counted in randomly selected B-cell rich areas in tissue sections (a minimum of five 0.15 mm^2^ image fields produced with a 20× objective per tissue per animal). The total numbers of CD79α^+^ cells in the areas analyzed were then estimated by dividing the total area of CD79α^+^ immunofluorescence with the average area in a single B lymphocyte in threshold-segmented images, using ImageJ [35]. The minimum number of B cells thus analysed per animal was 80 in fetal bone marrow, 2440 in lymph nodes, 900 in spleen, 1280 in fetal liver and 21500 in fetal Peyer's patch, reflecting the B cell densities in these tissues. For the tissues where no TdT^+^ B cells were observed, a minimum of 1200 B cells were screened per section (except for adult bone marrow and liver, where B cells are very rare).

## Results

### Data mining and sequencing uncovers potentially novel IGHV and IGHD genes

In order to ensure a complete set of reference germline immunoglobulin heavy chain gene sequences for analyses of junctional diversity, we sequenced the *IGHV* genes in the four animals used in this study and mined all available bovine genomic sequence data for *IGHD* genes.

In addition to the 10 functional *IGHV* genes previously annotated in the genomic data [36], we identified 26 new germline *IGHV* sequences. These were assigned temporary gene designations based on the IMGT nomenclature [37]. The 26 sequences have been deposited to GenBank as KJ491073-KJ491098 and are listed in Table S1. They all belong to the subgroup IGHV1 and include potentially new genes as well as new allelic variants of existing genes.

Data mining uncovered four new *IGHD* sequences (*IGHDS10* to *IGHDS13*, Table S2) in addition to the previously characterized 10 *IGHD* genes [7], [28]. Pairwise alignments between the new *IGHD* sequences and immunoglobulin cDNAs strongly suggested the presence of a fifth novel germline sequence *IGHDS14* that was related to *IGHDS12* (Figure S1). To deduce the *IGHDS14* sequence, multiple sequence alignment was done among the corresponding cDNAs that were derived from at least 20 different recombinations based on variable *IGHV* gene usage and CDR3H length. The consensus sequence representing the partial *IGHDS14* sequence was then determined (Figure S2, Table S2). The five novel *IGHD* sequences represent uncharacterized genes or allelic variants of existing genes. Their length ranged from 31 bp (*IGHDS10*) to 154 bp (*IGHDS12*), the longest of bovine *IGHD* genes identified to date. They were variably used in immunoglobulin gene recombinations (Table 2).

**Table 2:** Expressed combinations of IGHV, IGHD and IGHJ segments in bovine fetal bone marrow, ileum and spleen.

V gene	IGHDS5	IGHDS3	IGHDS7	IGHDS8	IGHDS2	IGHDS14	IGHDS4	IGHDS11	IGHDS12	IGHDS9	IGHDS1	IGHDS10	IGHDS13	IGHDS6	sum
**IGHV1S3**	31/0/2	19/0/0	24/0/1	16/0/13	3/0/5	8/0/0	11/0/0	4/0/0	0/0/0	1/0/0	3/0/0	1/0/0	0/0/0	1/0/0	143
**IGHV1S39**	84/0/0	2/0/0	8/0/1	0/0/3	1/0/0	1/0/0	0/0/0	2/0/0	1/0/0	0/0/0	0/0/0	0/0/0	0/0/0	0/0/0	103
**IGHV1S15**	25/0/0	32/0/0	5/0/0	9/0/0	13/0/0	0/0/0	7/0/0	1/0/0	0/0/0	2/0/0	1/0/0	1/0/0	3/0/0	0/0/0	99
**IGHV1S28**	27/0/0	8/0/0	10/0/0	2/0/0	10/0/0	2/0/0	3/0/0	0/0/0	1/0/0	0/0/1	0/0/0	0/0/0	0/0/0	0/0/0	64
**IGHV1S1**	14/0/0	3/0/1	3/0/0	2/0/9	2/0/1	17/0/0	0/0/0	0/0/0	5/0/0	0/0/0	0/0/0	0/0/0	0/0/0	0/0/0	57
**IGHV1S6**	15/0/0	3/0/0	4/0/0	0/0/0	0/0/0	0/0/0	1/0/0	0/0/0	0/0/0	0/0/0	0/0/0	0/0/0	0/0/0	0/0/0	23
**IGHV1S18**	15/0/0	4/0/0	2/0/0	0/0/0	0/0/0	0/0/0	0/0/0	0/0/0	0/0/0	0/0/0	0/0/0	0/0/0	0/0/0	0/0/0	21
**IGHV1S34**	11/0/0	6/0/0	1/0/0	1/0/0	1/0/0	0/0/0	0/0/0	0/0/0	1/0/0	0/0/0	0/0/0	0/0/0	0/0/0	0/0/0	21
**IGHV1S11**	6/0/0	3/0/2	4/0/0	0/0/1	0/0/0	3/0/0	0/0/0	0/0/0	0/0/0	0/0/0	0/0/0	1/0/0	0/0/0	0/0/0	20
**IGHV1S14**	2/0/0	1/0/0	5/0/0	2/0/3	1/0/0	1/0/0	1/0/0	0/0/0	0/0/0	1/0/0	0/0/0	0/0/0	0/0/0	0/0/0	17
**IGHV1S10**	8/0/0	2/0/0	3/0/0	0/0/0	1/0/0	0/0/0	0/0/0	0/0/0	1/0/0	0/0/0	0/0/0	0/0/0	0/0/0	0/0/0	15
**IGHV1S33**	6/0/0	1/0/0	4/0/0	1/0/0	0/0/0	0/0/0	0/0/0	1/0/0	0/0/0	0/0/0	0/0/0	0/0/0	0/0/0	0/0/0	13
**IGHV1S31**	9/0/0	1/0/0	0/0/0	0/0/0	0/0/0	0/0/0	0/0/0	1/0/0	0/0/0	0/0/0	0/0/0	0/0/0	0/0/0	0/0/0	11
**IGHV1S16**	4/0/0	0/0/0	1/0/0	0/0/0	0/0/0	0/0/0	0/0/0	0/0/0	0/0/0	0/0/0	0/0/0	0/0/0	0/0/0	0/0/0	5
**IGHV1S2**	2/0/0	1/0/0	0/0/0	0/0/2	0/0/0	0/0/0	0/0/0	0/0/0	0/0/0	0/0/0	0/0/0	0/0/0	0/0/0	0/0/0	5
**IGHV1S24**	0/1/0	0/0/0	0/0/0	0/0/0	0/0/0	0/0/0	0/0/0	0/0/0	0/0/0	4/0/0	0/0/0	0/0/0	0/0/0	0/0/0	5
**IGHV1S40**	3/0/0	0/0/0	0/0/0	0/0/0	2/0/0	0/0/0	0/0/0	0/0/0	0/0/0	0/0/0	0/0/0	0/0/0	0/0/0	0/0/0	5
**IGHV1S4**	0/0/0	1/0/0	0/0/0	0/0/3	0/0/0	0/0/0	0/0/0	0/0/0	0/0/0	0/0/0	0/0/0	0/0/0	0/0/0	0/0/0	4
**IGHV1S35**	1/0/0	0/0/0	1/0/0	0/0/0	1/0/0	0/0/0	0/0/0	0/0/0	0/0/0	0/0/0	0/0/0	0/0/0	0/0/0	0/0/0	3
**IGHV1S7**	1/0/0	1/0/0	0/0/0	1/0/0	0/0/0	0/0/0	0/0/0	0/0/0	0/0/0	0/0/0	0/0/0	0/0/0	0/0/0	0/0/0	3
**IGHV1S26**	0/0/0	0/0/0	0/0/0	0/0/1	0/0/0	0/0/0	1/0/0	0/0/0	0/0/0	0/0/0	0/0/0	0/0/0	0/0/0	0/0/0	2
**IGHV1S29**	1/0/0	0/0/0	1/0/0	0/0/0	0/0/0	0/0/0	0/0/0	0/0/0	0/0/0	0/0/0	0/0/0	0/0/0	0/0/0	0/0/0	2
**IGHV1S19**	0/0/0	0/0/0	1/0/0	0/0/0	0/0/0	0/0/0	0/0/0	0/0/0	0/0/0	0/0/0	0/0/0	0/0/0	0/0/0	0/0/0	1
**IGHV1S27**	0/0/0	0/0/0	0/0/0	1/0/0	0/0/0	0/0/0	0/0/0	0/0/0	0/0/0	0/0/0	0/0/0	0/0/0	0/0/0	0/0/0	1
**IGHV1S36**	0/0/0	1/0/0	0/0/0	0/0/0	0/0/0	0/0/0	0/0/0	0/0/0	0/0/0	0/0/0	0/0/0	0/0/0	0/0/0	0/0/0	1
**IGHV1S38**	0/0/0	0/0/0	0/0/0	0/0/1	0/0/0	0/0/0	0/0/0	0/0/0	0/0/0	0/0/0	0/0/0	0/0/0	0/0/0	0/0/0	1
**per JH1/2/6**	265/1/2	89/0/3	77/0/2	35/0/36	35/0/6	32/0/0	24/0/0	9/0/0	9/0/0	8/0/1	4/0/0	3/0/0	3/0/0	1/0/0	594/1/50
**Sum**	268	92	79	71	41(*)	32	24	9	9	9	4	3	3	1	645

A total of 645 cDNA sequences were analyzed from bone marrow, ileum and spleen. Each cell shows the numbers of cDNAs containing the *JH1*, *JH2* and *JH6* gene segment. The *IGHV* segment is specified by the row and the *IGHD* gene by the column. As an example, the top left cell shows that there were 31 cDNAs containing the combination *IGHV1S3-IGHDS5-JH1* and 2 cDNAs corresponding to *IGHV1S3-IGHDS5-JH6*. The most commonly expressed genes were *IGHV1S3*, *IGHDS5* and *JH1*. The most common combination was *IGHV1S39-IGHDS5-JH1*. (*) In 31 cDNAs, the ends of *IGHDS2* were extensively trimmed (Table 6).

### A limited range of gene combinations is found in the immunoglobulin cDNAs

cDNA libraries from fetal bone marrow (specific for *IGH*, *IGL*, *IGK*), ileum (*IGH*, *IGL*, *IGK*) and spleen (*IGH*) were analyzed for various combinations of immunoglobulin variable, diversity and joining genes (Tables 2 and 3). Twenty-six *IGHV* genes were found in the cDNA sequences (N = 645) of which five genes (*IGHV1S3*, *IGHV1S39*, *IGHV1S15*, *IGHV1S28* and *IGHV1S1*) accounted for 72%. Thirteen *IGHD* genes were detected in the cDNA sequences. *IGHDS5* (*DH5* [21]) was used in 42% and *IGHJS1* (*JH1* [27]) in 92% of the sequences. The most common combination was *IGHV1S39*-*IGHDS5*-*IGHJS1*, which occurred in 13% of all cDNAs analyzed. The long *IGHDS2* (148 bp), *IGHDS12* (154 bp) and *IGHDS14* (119 bp or longer) genes were found in 13% of the recombinations.

**Table 3:** Expressed combinations of *IGLV* and *IGLJ* gene segments in bovine fetal bone marrow and ileum.

	*IGLJ3*	*IGLJ2*
***IGLV30***	20	3
***IGLV39***	7	1
***IGLV49***	7	
***IGLV8***	6	1
***IGLV43***	4	
***IGLV28***	4	
***IGLV25***	3	
***IGLV55***	2	
***IGLV35***	2	
***IGLV2***	2	
***IGLV6***	2	
***IGLV56***		1

n = 65.

The immunoglobulin λ cDNA sequences from bone marrow and ileum of a 270 days old fetus matched to 12 of the previously identified 25 potentially functional *IGLV* genes [30]. All of these genes belong to subgroup 1. The preferential gene usage did not differ between the two tissues. *IGLV30* was the most common of the variable genes expressed, and the combination *IGLV30*-*IGLJ3* accounted for 35% of the cDNA sequences (Table 3). The second common variable gene used was *IGLV39* (12%). *IGLJ2* was used only in 9% of sequences whereas *IGLJ3* was used in 91%. We identified the expression of 3 *κ* variable genes out of the 8 potentially functional genes [30]. *IGKV19* was used in 64% (Table 4) whereas 35% of the sequences contained *IGKV10*. Also *IGKV17* was detected in 1% of the sequences. In 97% of the sequences, *IGKJ1* was used.

**Table 4:** Expressed combinations of *IGKV* and *IGKJ* gene segments in bovine fetal bone marrow and ileum.

	*IGKJ1*	*IGKJ2*
***IGKV19***	53	
***IGKV10***	28	1
***IGKV17***	1	

n = 83.

### N nucleotide additions and exonuclease activity shape the CDR3H region

**Figure 2:**
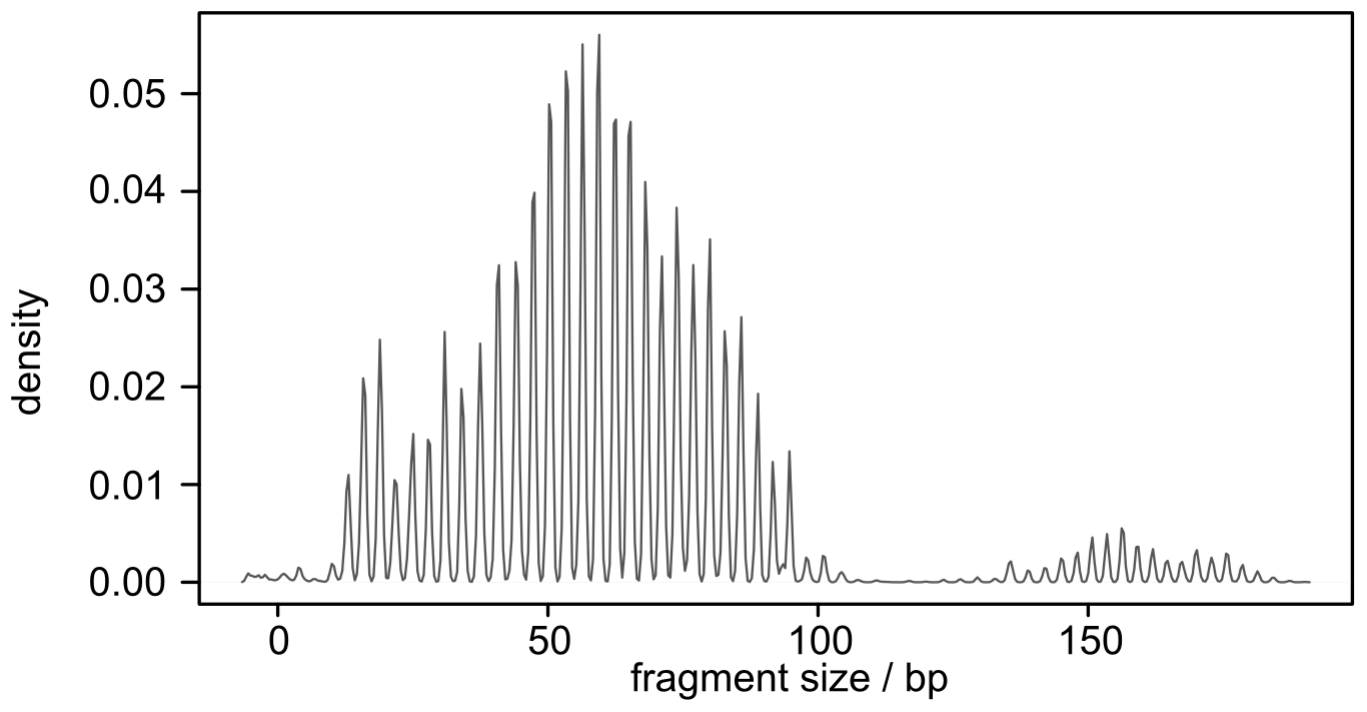
Bovine fetal IGH spectratype. Pooled data from 24 samples (four fetuses, six tissues/fetus: thymus, spleen, ileum, lymph node, liver, and bone marrow). Fragment size corresponds to the length of CDR3.

We analyzed the CDR3H encoding region in 645 cDNA sequences derived from bone marrow, ileum and spleen of two nearly full term fetuses (Tables S4 and S5). The average length of CDR3H encoding region in the recovered clones was 74.9 nucleotides. In 8.4% of the sequences the CDR3H encoding region was over 100 bp long suggesting a second subpopulation of bovine IGH cDNAs with long CDR3H encoding region [3]. This was confirmed by a separate spectratyping assessment of the CDR3H lengths of fetal thymus, spleen, ileum, lymph node, liver and bone marrow (Figure 2).

**Figure 3:**
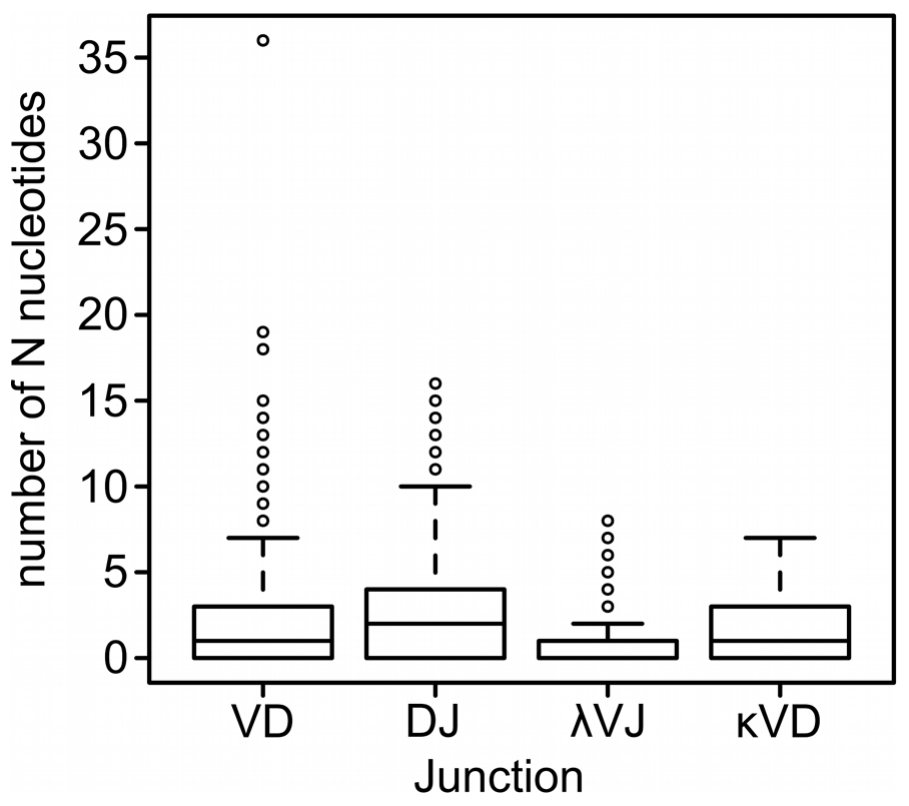
Junctional diversity in bovine fetuses. N nucleotide additions in IGH (VD and DJ junctions, n = 645) IGL (λVJ junction, n = 65) and IGK (κVJ junction, n = 83). Dark line in the middle represents the median and 50% of the cases lie within the box. Whiskers extend to 1.5 times the height of the box. Circles represent outliers. We did not detect statistically significant differences between tissues or individuals. Heavy chain data is pooled from bone marrow, ileum and spleen (two fetuses). Light chain data is pooled from bone marrow and ileum (one fetus).

The median of N nucleotides was 1 in VD and 2 in DJ junctions (n = 645, Figure 3). They were found in 65% in VD and 68% in DJ junctions. All in all, 90% of the sequences contained N nucleotides. Some extremely long N additions could also be seen. More than 10 N additions (range 10 to 36 nt) were found in 4.5% of VD junctions and 3.6% (range 10 to 16 nt) of DJ junctions. Palindromic P nucleotides were also seen, with 16% of VD junctions (range 1 to 6) and 18% of DJ junctions (range 1 to 3) showing P additions (Figure 3 and Table 5). The base profile of N nucleotide additions in VD junctions was dominated by T (33%) and A (31%) followed by G (19%) and C (17%). The profile was more homogenous in the DJ junctions with about equal frequency of T and A (26% each) vs. G (28%) and C (21%). We could not detect conserved short nucleotide sequences (CSNS) that have previously been reported in adult bovine VDJ recombinations [7].

**Table 5:** Analysis of nucleotide additions in bovine fetal IGH (bone marrow), IGL and IGK (bone marrow and ileum).

Junction	Number of sequences	Median number of N nucleotides (range)	Sequences with N additions (%)	Long (>10) N additions (%)	Median number of P nucleotides (range)	Sequences with P additions (%)
**VD**	645	1 (0–36)	65	4.5	0 (0–6)	16
**DJ**	645	2 (0–16)	68	3.6	0 (0–3)	18
**VJ λ**	65	0 (0–8)	36.9	0	0 (0–1)	2
**VJ κ**	83	1 (0–7)	60	0	0 (0–1)	2

As the readout for the exonuclease activity, we used the loss of nucleotides from the ends of V, D, and J gene segments. The exonuclease activity removed a median of 2 nucleotides from the 3′ end of *IGHV* and from the 5′ end of *IGHJ*. We also detected extensive trimming of *IGHD* gene ends. The median value of the number of deleted nucleotides from the ends of *IGHD* genes was 5 in a VD junction and 6 in a DJ junction. There was a statistically significant difference between the number of deleted nucleotides from D genes vs. V or J genes (Mann-Whitney U test, *P*<1e-16).

### N-nucleotide additions occur in λ and κ light chains but to a lesser extent than in the heavy chains

We sequenced 65 IGL and 83 IGK cDNA clones from bone marrow and ileum of the same fetuses as above. The numbers of N additions found in VJ junctions were similar between the two tissues. Therefore, we pooled the results from bone marrow and ileum. Nontemplated additions were found in 36.9% (24) of IGL clones and 60% (50) of IGK clones. The median of N nucleotides was 0 in IGL and 1 in IGK (Figure 3). There was a statistically significant difference between the number of N additions in IGL and IGK (Mann-Whitney U test; *P* = 0.006). Very few P nucleotides could be detected in light chains (Table 5). Exonuclease activity was also detected in light chains: a median value of 2.5 nucleotides was excised from the 3′ end of *IGLV* and *IGKV* genes. The corresponding numbers in the 5′ end of joining genes were as follows: in *IGLJ* 1 bp and in *IGKJ* 3 bp (Mann-Whitney U test *P*<7e-08). As in the heavy chain sequences, the junctional base profile was dominated by T (36.4%) and A (24.3%) followed by C (20.4%) and G (19.0%).

### TdT and its splice variants are expressed in bone marrow in bovine fetuses

**Figure 4:**
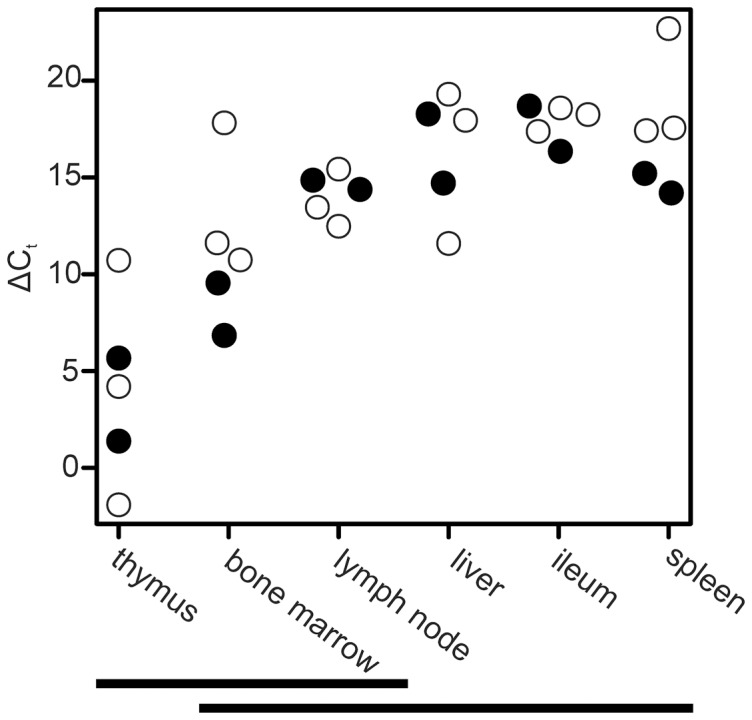
TdT mRNA expression level in adults and fetuses. The expression level was measured with RT-qPCR. Material consisted of 3 fetuses and 2 adults. Thymus, bone marrow and lymph node had elevated expression levels compared to liver, ileum and spleen. Fetuses did not differ from adults. 18S normalized cycle threshold values (ΔC_t_) are shown. Tissues not differing statistically (α = 0.05) from each other are indicated by a horizontal bar. White points indicate fetuses and black points indicate adults.

The expression of TdT mRNA was measured with RT-qPCR in 3 fetal and 2 adult cattle. The general primers located to exon 2 and can also amplify the long isoforms (Figure 1). Thymus, bone marrow and lymph node showed elevated expression levels compared to liver, ileum and spleen (Figure 4, *P* = 0.003, α = 0.05). Fetuses did not differ from adults.

Expression of known TdT isoforms, bovineTdTL1 and bovineTdTL2, was also assessed with RT-qPCR. The long isoforms L1 and L2 both contain an extra exon VI’ and X’ respectively (Figure 1). The highest expression levels were seen in thymus. In fetuses, the expression of both L1 and L2 differed between thymus and spleen and between thymus and ileum (P<0.0004, α = 0.01, data not shown). There were no statistical differences between the levels of tissue specific expression of either long isoform in adults (data not shown).

**Figure 5:**
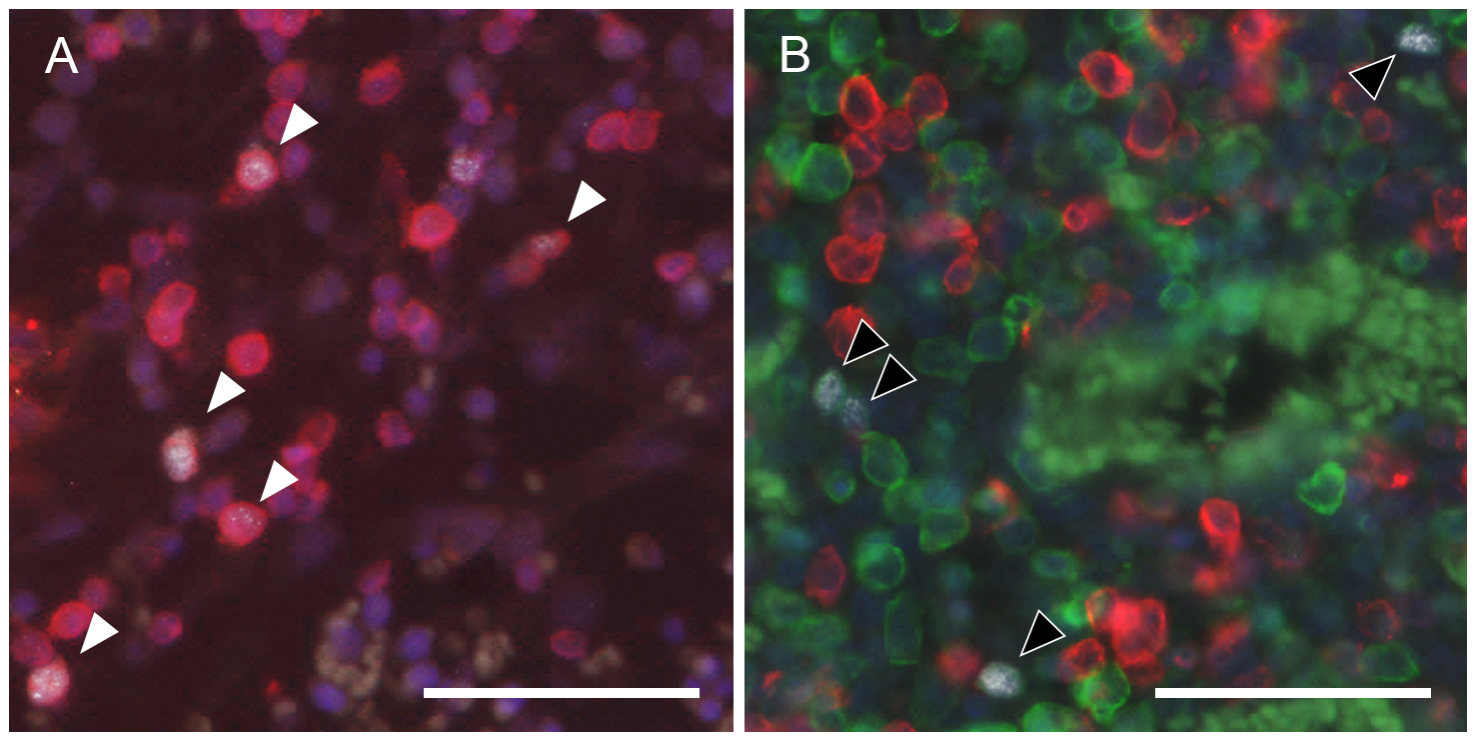
Immunofluorescence staining of fetal bovine bone marrow (A) and lymph node (B). Red: B lymphocyte marker CD79α. Green: T lymphocyte marker CD3. White: TdT. Blue: DAPI. White arrows: TdT positive B cells. Black arrows: TdT positive T cells. Scale bar: 50 µm.

To identify the cell types expressing TdT in various tissues, we performed triple immunofluorescence for TdT, the B lymphocyte marker CD79α and the T lymphocyte marker CD3. In the fetal bone marrow, 41±13% of the TdT positive cells were identified as CD79α^+^CD3^−^ B lymphocytes and 8.2±2.9% as CD3^+^ T lymphocytes (n = 5, average ± SD; Figure 5A). Of all bone marrow CD79α^+^ B lymphocytes, 11±2.5% expressed TdT. In contrast, in the fetal lymph node, 29±18% of the TdT positive cells were B lymphocytes, 33±14% were T lymphocytes, and only 0.14±0.08% of the B lymphocytes expressed TdT (n = 4; Figure 5B). Fetal spleen, liver and ileal Peyer's patch, as well as adult lymph node and spleen contained very few TdT^+^ B cells (0.002–0.04% of all CD79α^+^ B cells). B lymphocytes were rare in adult bone marrow and liver.

## Discussion

To complement their restricted range of immunoglobulin genes [29], [30], [38], [39], fetal cattle use two main mechanisms to secure a functional preimmune antibody repertoire: AID-driven somatic hypermutation [6] and TdT-mediated junctional diversity that is the focus of this paper. We first searched for potentially new immunoglobulin genes, as assessing junctional diversity is dependent on the accurate definition of V, D, and J gene segment boundaries in the rearranged immunoglobulin genes. We then analyzed the junctions between V, D, and J segments. Finally, we characterized the expression of TdT and its isoforms in fetal tissues.

### Bovine immunoglobulin heavy chain variable and diversity genes

We complied with the IMGT recommendations [37] for naming the immunoglobulin genes (Tables S1 and S2). *IGHV1S37* has been previously published [40] with accession JN897034, and several of the other sequences differed from the previously published ones by one nucleotide only. To ease the comparisons with the previously identified *IGHD* genes, all the currently known *IGHD* genes were summarized (Table S1) and the old gene names (*DH1* to *DH5*, *D64*, *DH7*, *DH8*, and *DQ52*) [7], [28] were retained next to those complying with IMGT recommendations (*IGHDS1* to *IGHDS9* and *IGHDS10* to *IGHDS14* corresponding to the previously known and new *IGHD* genes, respectively).

Twenty-six new potentially functional *IGHV* genes representing a single subgroup were identified from muscle genomic DNA (Table S1). Together with the previously reported 10 functional genes [36], the total number of potentially functional *IGHV* genes in our material was 36 of which a maximum of 20 were found from a single animal. However, these gene sequences represent both actual paralogous genes and allelic variants, which cannot be distinguished only based on gene sequence data [41]. As there is a maximum of two alleles per locus in a diploid genome, these observations suggest that cattle have 10 to 20 functional paralogous *IGHV* genes in total (presuming 100% heterozygosity or 100% homozygosity, respectively).

Long CDR3Hs and long *IGHD*s are well documented in cattle immunoglobulins [3], [5], [7], [42]. In our data from fetal bone marrow, ileum and spleen, the long *IGHD* genes (>100 nucleotides) were utilized in 13% of the recombinations. It was recently proposed that the domains of “ultra long” CDR3Hs form a unique antibody structure. The authors also described the longest CDR3H to date with 67 codons [43]. The CDR3H encoding regions in our material ranged from 5 to 65 codons. The longest CDR3Hs were encoded by *IGHV1S1* or *IGHV1S15*, *IGHDS12* or *IGHDS2* and *JH1*. *IGHV1S1* and *IGHV1S15* code for an unusual “TTVHQ” terminal motif, which initiates an ascending β strand in the folded antibody [43]. To date, these long CDR3H populations have not been detected in sheep or swine [44], [45].

To uncover new bovine *IGHD* genes we searched the entire high throughput genomic and trace archive databases at NCBI using RSS motives as queries. The utilized RSS motives identify all published bovine RSS sequences and also most of the published human RSSs. The novel *IGHDS14* gene uncovered from the sequenced cDNAs could not be found in the archives. It is possible that additional *IGHD* genes or allelic variants are present in the archives and thus remain to be discovered. The correct assignment of a particular D segment to cDNAs with a short CDR3H by best pairwise alignment score is further compromised by the short sequence motifs shared by several D segments (Table S2).

### Size variation in bovine immunoglobulin heavy and light chain N regions

We detected additions of several N nucleotides in most fetal *IGH* cDNA sequences suggesting that TdT is active in fetal life. Exceptionally long (10–36 bp) N nucleotide additions were detected in 4.0% of VD and 3.2% of DJ junctions. Such long N additions have not been reported in other species to our knowledge. In contrast, N regions longer than 10 nucleotides are considered abnormal in mice [46], [47]. Nontemplated additions to *IGH* genes have been reported in fetal humans [48], swine [44], [49] and sheep [45] but not in fetal or neonatal mouse [50]. In humans 1-6.9 N nucleotides are added on average, depending on the *IGHD* gene used [48]. Also, N additions in fetal swine are fairly common. Average number is 7.9–9.9 nucleotides and can reach up to 20 nucleotides per coding joint [44], [49]. In sheep, the exact analysis of the junctional variability has not been possible because of the lack of knowledge of the *IGHD* genes [45]. Despite the great range of N additions in our data, their average number in cattle was not especially high (2.5/VD junction and 2.6/DJ junction) compared to other species, reflecting the high frequency (35%) of junctions with zero additions.

Nontemplated nucleotide additions were also present in *IGL* and *IGK* light chain genes although their number was lower than in heavy chain genes. Junctional diversity has been observed in the ovine IGK light chains. In ovine *IGL* genes, the extent of junctional diversity appears to depend on the specific joining gene used [51], [52]. Very little junctional diversity is seen in swine light chains [53], [54]. In human and mouse junctional diversity is absent in light chains, as TdT expression is restricted to the pro-B stages. The expression starts to weaken already during pre-B cell stage and is absent in later stages when light chain rearrangements occur [55], [56].

In addition to N-nucleotide additions, the junctional regions were modified by exonuclease activity targeted to *IGL* and *IGH* gene ends. The median number of removed nucleotides ranged from 1 to 3 for the variable and joining gene ends. In contrast, the trimming of the *IGHD* gene ends appeared more extensive (median value of 5 and 6 nucleotides for the VD and JD junction, respectively). Extensive trimming of *IGHD* genes is also observed in swine where the longer porcine D_H_A was trimmed to the same length as the shorter D_H_B [44]. Protein conformation tolerates the trimming of *IGHD* better than that of *IGHV* or *IGHJ*, because *IGHD* does not encode for framework regions. In our data, there were 63 (10%) cDNAs where the number of removed nucleotides from either junction was greater than 29. Removal of these cDNAs did not affect the median or range of P or N nucleotide additions presented in Table 2. However, *IGHDS2* was assigned to 31 of these cDNAs (Table 6). The frequency of *IGHDS2* in recombinations (Table 2) has to be interpreted with great caution.

**Table 6:** The effect of removal of 63 cDNAs linked to high exonuclease activity to the frequency of *IGHD* segments in bovine fetal immunoglobulin cDNAs.

IGHD	High exo	Normal exo	Sum
**IGHDS1**	1	3	4
**IGHDS2**	31	10	41
**IGHDS3**	7	85	92
**IGHDS4**	0	24	24
**IGHDS5**	14	254	268
**IGHDS6**	0	1	1
**IGHDS7**	0	79	79
**IGHDS8**	0	71	71
**IGHDS9**	0	9	9
**IGHDS10**	0	3	3
**IGHDS11**	5	4	9
**IGHDS12**	2	7	9
**IGHDS13**	0	3	3
**IGHDS14**	3	29	32
**Sum**	**63**	**582**	**645**

Exonuclease activity was deduced from the alignments with the best matching *IGHD* segment and quantified by the number of apparently excised nucleotides. High exonuclease activity: excision of over 29 nucleotides from either end of the *IGHD* segment.

We defined the D-region boundaries on the basis of the coordinates of the best pairwise alignment between the cDNA sequence and *IGHD* genes. To ensure that only nontemplated and P nucleotides are included in the VD and DJ joints, the extension of gaps was only marginally penalized. The presence of gaps in the alignments may indicate that the existence of additional *IGHD* genes or alleles. This will not affect the overall conclusions of this work since the total number of sequences with gaps in the alignment was 11 (2%). Alternatively, SHM process might induce small insertions and deletions. Also, *IGHD* genes contain repetitive TAT and GGT codons that could be incorrectly copied by the cellular DNA polymerases either during SHM or DNA replication.

### TdT expression in fetal cattle

We analysed the fetal expression of TdT while adult thymus served as a positive control [57], [58]. Of extra-thymic tissues, bone marrow displayed the strongest TdT expression with more than 10% of all B cells being TdT positive. TdT expression was also detected in fetal lymph nodes, but this was largely due to TdT positive T cells; only a very small fraction of lymph node B cells expressed TdT (Figures 4 and 5). A significant number of TdT positive cells were negative for both CD markers. These possibly represent lymphoid progenitor cells, which sometimes express TdT already at the CD34^+^ stage [59]. In fact, TdT was originally considered a marker for immature lymphoid cells [60]. TdT expression has also been shown to be associated with acute myeloid leukemia, suggesting that TdT expression is not always limited to cells fully committed to the lymphoid lineage [61], [62]. This may also explain our finding that TdT expression, as measured by qPCR, is at a similar level in the adult and fetal bone marrow as shown in Figure 4. In adults, B cells are very rare and *de novo* B lymphopoiesis has practically ceased [23].

Alternative splicing occurs in TdT. Long splice variants (TdTLs), which possess an extra exon forming a new catalytic site for the enzyme, have been suggested to have exonuclease activity in humans [21]. Like humans, cattle have three potential isoforms. In addition to the shorter form (bTdTS), two longer fragments (bTdTL1 and bTdTL2) have been found from bovine thymic cDNA [20]. We detected the long variants mainly in thymus while the expression levels in other tissues were low. This suggests that long isoforms could be mainly T-cell specific in cattle.

### TdT and N-nucleotide additions in fetal cattle

TdT is sufficient for N-region diversity in mouse immunoglobulin loci [46]. However, the very long N additions observed here differ from the murine TdT signature [46], [47]. Also, the bias towards T additions is in contrast to the previous findings of 60–70% of dGMP residues in N additions *in vitro* [13]. There are plausible explanations for these differences. First, the G/C nucleotide bias is less emphasized in long extensions. The dGMP nucleotides tend to form aggregates resulting in the 3′-OH group of the growing polymer becoming relatively less accessible to further chain growth [63], [64]. This suggests that long G/C rich additions may be disfavored *in vivo* due to conformational restrictions. Second, the function of TdT is known to be dissimilar *in vivo* versus *in vitro*. Mouse *in vivo* studies show 2–5 nucleotide additions [65] while *in vitro* TdT can add several kilobases of nucleotides under optimal conditions [12]. DNA-PK limits the length of TdT-induced nucleotide additions *in vitro* by reducing the number of modified DNA ends and the length of nucleotide additions [64]. More recently, Ku80, which is a part of DNA-PK, was shown to inhibit the DNA strand elongation activity by TdT [47]. DNA-PK or Ku80 proteins have not been investigated in cattle so it remains to be resolved whether or not these components are involved in regulating the bovine TdT activity. The lack a TdT inhibitor would give better access to the free coding ends during V(D)J recombination, promote more efficient initiation of polymerization and lead to a greater number of modified V(D)J ends. Alternatively, it could permit longer nucleotide additions than seen in human or mouse by increasing the processivity of TdT.

In addition to TdT, other polymerases of the PolX family may also contribute to junctional diversity. Polµ deficient mice have about 6 bp shorter VJ junctions in κ light chains compared to wild type, suggesting that Polµ takes part in immunoglobulin gene diversification after TdT expression has decreased [66]. In mice Polµ is active during early embryonic DJ_H_ rearrangements. It can perform template-independent nucleotide additions in a similar manner to TdT [16]. Also Polλ polymerase functions in heavy chain rearrangements [67]. Apart from TdT, other PolX family members are currently uncharacterized in cattle and require further investigation.

In conclusion, our data suggest that junctional diversity plays a significant role in the generation of the bovine preimmune immunoglobulin repertoire. TdT is expressed in fetal bone marrow B cells in conjunction with the recombination machinery [22], [23]. The analysis of immunoglobulin cDNA sequences confirms the diversification of the V(D)J junctions by a combination of polymerase and exonuclease activities. According to the prevailing model of B-cell development in ruminants [2], a subpopulation of B cells expressing the B cell receptor then seeds the ileal Peyer's patch where somatic hypermutation further diversifies the repertoire. Together, junctional diversity and somatic hypermutation complement the small range of immunoglobulin genes and enable the creation of a sufficiently large functional preimmune repertoire during late fetal life.

## Supporting Information

Figure S1 **Alignment views of immunoglobulin heavy chain sequences with** ***IGHD*** **gene segments.** Initial alignments of 645 immunoglobulin cDNAs to the best matching reference gene from *IGHDS1* to *IGHDS13*. Boundaries of the CDR3H region as defined by IMGT and putative amino acid sequence are indicated. Note that many of the cDNAs matching to *IGHDS12* contain a novel gene segment *IGHDS14* (see also Figure S2).
(TXT)

Figure S2 **Deduction of novel** ***IGHDS14*** **sequence.** A set of cDNA sequences was selected based on pairwise alignments with *IGHDS12* in Figure S1. Regions between V and J segments were extracted and aligned with MUSCLE [26]. Consensus sequence corresponding to *IGHDS14* is shown. Star (*) indicates a completely conserved nucleotide.
(TXT)

Table S1 **New bovine IGHV sequences characterized in this study.** The sequences have been submitted to GenBank (accessions KJ491073-KJ491098). *IGHVS18-IGHVS40* contain first eight bases of RSS (typed in lower case).
(DOCX)

Table S2 **General information on bovine gene segments IGHDS1-IGHDS14.**
(XLSX)

Table S3 **General information on bovine gene segments JH1, JH2 and JH6.**
(XLSX)

Table S4 **Sequence analysis of CDR3H region.** Nucleotides corresponding to V, D and J gene segments, N and P nucleotides in VD and DJ junctions, and putative peptide sequence are shown for 645 fetal bovine immunoglobulin cDNAs. Small letters: framework nucleotides. Capital letters: nucleotides corresponding to CDR3H region.
(XLSX)

Table S5 **Quantification of N and P nucleotide additions and exonuclease activity during immunoglobulin recombination at the heavy chain locus.** The VDJ junctions of 645 bovine fetal immunoglobulin cDNAs were analyzed from pairwise alignments with best matching reference V, D, and J gene segments. The number of added (N and P) nucleotides and the number of removed nucleotides from each reference gene end are shown.
(XLSX)

Table S6 **Quantification of N and P nucleotide additions and exonuclease activity during immunoglobulin recombination and the λ light chain locus.** The VJ junctions of 65 bovine fetal λ light chain cDNAs were analyzed from pairwise alignments with best matching reference V and J gene segments. The number of added (N and P) nucleotides and the number of removed nucleotides from each reference gene end are shown.
(XLSX)

Table S7 **Quantification of N and P nucleotide additions and exonuclease activity during immunoglobulin recombination at the κ light chain locus.** The VJ junctions of 83 bovine fetal κ light chain cDNAs were analyzed from pairwise alignments with best matching reference V and J gene segments. The number of added (N and P) nucleotides and the number of removed nucleotides from each reference gene end are shown.
(XLSX)
