# Supplementary material for: Expansion of the Preimmune Antibody Repertoire by Junctional Diversity in Bos taurus
Source: PLoS One. 2014 Jun 13;9(6):e99808. doi: 10.1371/journal.pone.0099808 (PMC4057420; doi:10.1371/journal.pone.0099808)
Supplement: Table S1 — New bovine IGHV sequences characterized in this study. The sequences have been submitted to GenBank (accessions KJ491073-KJ491098). IGHVS18-IGHVS40 contain first eight bases of RSS (typed in lower case). (DOCX) [file pone.0099808.s003.docx]

**Supplementary Table S1**. New bovine *IGHV* gene sequences characterized in this study. These sequences have been submitted to GenBank™ (accessions KJ491073-KJ491098). IGHVS18-IGHVS40 contain first eight bases of RSS (typed in lower case).

>IGHV1S15

CAGGTGCAGCTGCGGGAGTCGGGCCCCAGCCTGGTGAAGCCGTCACAGACCCTCTCCCTCACCTGCACGGTCTCTGGATTCTCATTGAGCGACAAGGCTGTAGGCTGGGTCCGCCAGGCTCCAGGGAAGGCGCTGGAGTGGCTCGGTGGTATAGACACTGGTGGAAGCACAGGCTATAACCCAGGCCTGAAATCCCGGCTCAGCATCACCAAGGACAACTCCAAGAGCCAAGTCTCTCTGTCAGTGAGCAGCGTGACAACTGAGGACTCGGCCACATACTACTGTACTACTGTGCACCAGA

>IGHV1S16

CAGGTGCAGCTGCGGGAGTCGGGCCCCAGCCTGGTGAAGCCGTCACAGACCCTCTCGCTCACCTGCACGGCCTCTGGATTCTCATTAAGCGACAAGGCTGTAGGCTGGGTCCGCCAGGCTCCAGGAAAGGCGCTGGAGTGGCTCGGTGGTATAGACACTGGTGGAAGCACAGGCTATAACCCAGGCCTGAAATCCCGGCTCAGCATCACCAAGGACAACTCCAAGAGCCAAGTCTCTCTGTCAGTGAGCAGCGTGACAACTGAGGACTCGGCCACATACTACTGTACTACTGTGCACCAGA

>IGHV1S17

CAGGTGCAGCTGCGGGAGTCGGGCCCCAGCCTGGTGAAGCCCTCACAGACCCTCTCCCTCACCTGCACGGCCTCTGGATTCTCATTAAGCGACAAGGCTGTAGGCTGGGTCCGCCAGGCTCCAGGAAAGGCGCTGGAGTGGCTCGGTGGTATAGACACTGGTGGAAGCACAGGCTATAACCCAGGCCTGAAATCCCGGCTCAGCATCACCAAGGACAACTCCAAGAGCCAAGTCTCTCTGTCAGTGAGCAGCGTGACAACTGAGGACTCGGCCACATACTACTGTACTACTGTGCACCAGA

>IGHV1S18

CAGGTGCAGCTGCGGGAGTCGGGCCCCAGCCTGGTGAAGCCCTCACAGACCCTCTCCCTCACCTGCACGGTCTCTGGATTCTCATTGAGCAGCAATGGTGTAGGCTGGGTCCGCCAGGCTCCAGGGAAGGCGCTGGAGTGGGTTGGTGGTATAGATAATGATGGAGACACATACTATAACCCAGCCCTGAAATCCCGGCTCAGCATCACCAAGGACAACTCCAAGAGCCAAGTCTCTCTGTCAGTGAGCAGCGTGACACCTGAGGACACGGCCACATACTACTGTGCGAAGGAcacagtga

>IGHV1S19

CAGGTGCAGCTGCAGGAGTCGGGCCCCAGCCTGGTGAAGCCCTCACAGACCCTGTCCCTCACCTGCACGGTCTCTGGATTCTCATTAAGCGACAATGCTGTAGGCTGGGTCCGCCAGGCTCCAGGAAAGGCGCTGGAGTAGCTTGGTGGTATAGATAATGATGGATACACAAGGTATAACCCAGCCCTGAAATCCCGGCTCAGCATTACCAAGGACAACTCCAAGAGCCAAGTCTCTCTGTCAGTGAGCAGCGTGACACCTGAGGACACGGCCACATACTACTGTGCAAAAGAcacagtga

>IGHV1S20

CAGGTGCAGCTGCGGGAGTCGGGCCCCAGCCTGGTGAAGCCCTCACAGACCCTCTCCCTCACCTGCACGGTCTCTGGATTCTCATTAAGCGATAATAGTGTAGGCTGGGTCCGCCAGGCTCCAGGAAAGGCGCTGGAGTGGCTCGGTGTCATATATAGTGGTGGAAGCACAGGCTATAACCCAGCCCTGAAATCCCGGCTCAGCATCACCAAGGACAACTCCAAGAGCCAAGTCTCTCTATCACTGAGCAGCGTGACAACTGAGGACACGGCCACATACTACTGTGCAAGAGAcacagtga

>IGHV1S21

CAGGTGCAGCTACAGGAGTCGGGCCCCAGCCTGGTGAAGACCTCACAGACACTCTCCCTCACTTGCACGGCCTCTGGATTATCATTAACCAGATATGGTATACACTGGGTCCGCCAGGCTCCAGGAAAGGCGCTGGAGTGGCTTGGTGATATAAGCAGTGGTGGAAGCACAGGCTATAACCCAGGCCTGAAATCCCGGCTCAGCATCACCAAGGACAACTCCAAGAGCCAAGTCTCTCTGTCACTGAGCAGCCTGACGCCTGAGGACTCAGCCACATACTACTGTGCAAGAGAcacagtga

>IGHV1S22

CAGGTGCAGCTGCGGGAGTCGGGCCCCAGCCTGGTGAAGCCCTCACAGACCCTCTCCCTCACCTGCACGGTCTCTGGATTCTCATTGAGCAGCAATGGTGTAGTCTGGGTCCGCCAGGCTCCAGGGAAGGCGCTGGAGTGGCTCGGTGGTATATGCAGTGGTGGAAGCACAAGCCTTAACCCAGCCCTGAAATCCCGGCTCAGCATCACCAAGGACAACTCCAAGAGCCAAGTCTCTCTGTCAGTGAGCAGCGTGACACCTGAGGACACGGCCACATACTACTGTGCAAGAGAcacagtga

>IGHV1S23

CAGGTGCAGCTGGGGGAGTCGGGCCCCAGCCTGGTGAAGCCCTCACAGACCCTCTCCCTCACCTGCACGACCTCTGGATTCTCATTGACCAGCTATGGTGTAAGCTGGGTCCGCCAGGCTCCAGGAAAGGCGCTGGAGTGGCTCGGTGGTATAGATAGTGGTGGAAGCACAGGCTATAACCCAGGCCTGAAATCCAGGCTCAGCATCACCAGGGACAACTCCAAGAGCCAAGTCTCTCTGTCAGTGAGCAGTGTGACACCTGAGGACACGGCCGTGTACTACTGTGCGAAGGAcacagtga

>IGHV1S24

CAGGTGCAGCTGCGGGAGTCGGGCCCCAGCCTGGTGAAGCCCTCACAGACCCTCTCCCTCACCTGCACGGCCTCTGGGTTCTCATTGAGCAGCTATGCTGTAAGCTGGGTCCGCCAGGCTCCAGGAAAGGCGCTGGAGTGGCTCGGTGGTATAGATACTGGTGGAAGCACAGGCTATAACCCAGGCCTGAATTCCCGGCTCAGCATCACCAAGGACAACTCCAAGAGCCAAGTCTCTCTGTCAGTGAGCAGCGTGACAACTGAGGACACGGCCGTGTACTACTGTGCGAAGGAcacagtga

>IGHV1S25

CAGGTGCAGCTGCGGGAGTCAGGCCCCAGCCTGGTGAAGCCCTCACAGACCCTCTCCCTCACCTGCACGACCTCTGGATTCTCATTGACCAGCTATGGTGTAAGCTGGGTCCGCCAGGCTCCAGGGAAGGCGCTGGAGTGGCTCGGTGGTATAGATAGTGGTGGAAGCACAGGCTATAACCCAGGCCTGAAACCCAGGCTCAGCATCACCAGGGACAACTCCAAGAGCCAAGTCTCTCTGTCAGTGAGCAGTGTGACACCTGAGGACACGGCCGTGTACTACTGTGCGAAGGAcacagtga

>IGHV1S26

AAGGTGCAGCTGCAGGAGTCGGGCCCCAGCCTGGTGAAGCCCTCACAGACCCTCTCCCTCACCTGCACGACCTCTGGATTCTCATTGACCAGCTATGGTGTAAGCTGGGTCCGCCAGGCTCCAGGAAAGGCGCTGGAGTGGCTCGGTGGTATAGATAGTGGTGGAAGCACAGGCTATAACCCAGGCCTGAAATCCAGGCTCAGCATCACCAGGGACAACTCCAAGAGCCAAGTCTCTCTGTCAGTGAGCAGCGTGACACCTGAGGACACGGCCGTGTACTACTGTGCGAAGGAcacagtga

>IGHV1S27

AAGGTGCAGCTGCAGGAGTCGGGCCCCAGCCTGGTGAAGCCCTCACAGACCCTCTCCCTCACCTGCACGACCTCTGGATTCTCATTGACCAGCTATGGTGTAAGCTGGGTCCGCCAGGCTCCAGGAAAGGCGCTGGAGTGGCTCGGTGGTATAGATAGTGGTGGAAGCACAGGCTATAACCCAGGCCTGAAATCCAGGCTCAGTATCACCAGGGACAACTCCAAGAGCCAAGTCTCTCTGTCAGTGAGCAGCGTGACACCTGAGGACACGGCCGTGTACTACTGTGCGAAGGAcacagtga

>IGHV1S28

CAGGTGCAGCTGCGGGAGTCAGGCCCCAGCCTGGTGAAGCCCTCACAGACCCTCTCCCTCACCTGCACGGTCTCTGGATTCTCATTGAGCAGCTATGCTGTAGGCTGGGTCCGCCAGGCTCCGGGGAAGGCACTGGAGTGGGTTGGTGGTATAAGTAGTGGTGGAAGCACATACTATAACCCAGCCCTGAAATCCCGGCTCAGCATCACCAAGGACAACTCCAAGAGCCAAGTCTCTCTGTCAGTGAGCAGCGTGACACCTGAGGACACGGCCACATACTACTGTGCAAAAGAcacagtga

>IGHV1S29

CAGGTGCAGCTGCGGGAGTCGGGCCCCAGCCTGGTGAAGCCCTCACAGACCCTCTCCCTCACCTGCACGATCTCTGGATTCTCATTGAGCAGCTATGCTGTAGGCTGGGTCCGCCAGGCTCCGGGGAAGGCGCTGGAGTGGGTTGGTGGTATAAGTAGTGGTGGAAGCACATGCCTTAACCCAGCCCTGAAATCCCGGCTCAGCATCACCAAGGACAACTCCAAGAGCCAAGTCTCTCTGTCAGTGAGCAGCGTGACACCTGAGGACACGGCCACATACTACTGTGCGAAGGAcacagtga

>IGHV1S30

CAGGTGCAGCTGCGGGAGTCAGGCCCCAGCCTGGTGAAGCCCTCACAGACCCTCTCCCTCACCTGCACGGTCTCTGGATTCTCATTGAGCAGCTATGCTGTAGGCTGGGTCCGCCAGGCTCCGGGGAAGGCACTGGAGTGGGTTGGTGGTATAGATAGTGGTGGAAGCACAGGCTATAACCCAGGCCTGAAATCCAGGCTCAGCATCACCAGGGACAACTCCAAGAGCCAAGTCTCTCTGTCAGTGAGCAGCGTGACACCTGAGGACACGGCCGTGTACTACTGTGCGAAGGAcacagtga

>IGHV1S31

CAGGTGCAGCTGCGGGAGTCGGGCCCCAGCCTGGTGAAGCCCTCACAGACCCTCTCCCTCACCTGCACGGTCTCTGGATTCTCATTGAGCAGCTATGCTGTAGGCTGGGTCCGCCAGGCTCCGGGGAAGGCGCTGGAGTGGGTTGGTGGTATAAGTAGTGGTGGAAGCACATGCCTTAACCCAGCCCTGAAATCCCGGCTCAGCATCACCAAGGACAACTCCAAGAGCCAAGTCTCTCTGTCAGTGAGCAGCGTGACACCTGAGGACACGGCCACATACTACTGTGCGAAGGAcacagtga

>IGHV1S32

CAGGTGCAGCTGCGGGAGTCAGGCCCCAGCCTGGTGAAGCCCTCACAGACCCTCTCCCTCACCTGCACGGTCTCTGGATTCTCATTGAGCAGCTATGCTGTAGGCTGGGTCCGCCAGGCTCCGGGGAAGGCACTGGAGTGGGTTGGTGGTATAAGTAGTGGTGGAAGCACATACTATAACCCAGCCCTGAAATCCCGGCTCAGCATCACCAGGGACAACTCCAAGAGCCAAGTCTCTCTGTCAGTGAGCAGCGTGACACCTGAGGACACGGCCACATACTACTGTGCAAAAGAcacagtga

>IGHV1S33

CAGGTGCAGCTGCGGGAGTCGGGCCCCAGCCTGGTGAAGCCCTCACAGACCCTCTCCCTCACCTGCACGGTCTCTGGATTCTCATTGAGCAGCTATGCTGTAAGCTGGGTCCGCCAGGCTCCAGGGAAGGCGCTGGAGTGCCTCGGTGGTATAAGCAGTGGTGGAAGCACAGGCTATAACCCAGCCCTGAAATCCCGGCTCAGCATCACCAAGGACAACTCCAAGAGCCAAGTCTCTCTGTCAGTGAGCAGCGTGACACCTGAGGACACGGCCACATACTACTGTGCAAGAGAcacagtga

>IGHV1S34

CAGGTGCAGCTGCGGGAGTCGGGCCCCAGCCTGGTGAAGCCCTCACAGACCCTCTCCCTCACCTGCACGGTCTCTGGATTCTCATTGAGCAGCTATGCTGTAAGCTGGGTCCGCCAGGCTCCAGGGAAGGCGCTGGAGTGGGTTGGTGGTATAAGCAGTGGTGGAAGCACATACTATAACCCAGCCCTGAAATCCCGGCTCAGCATCACCAAGGACAACTCCAAGAGCCAAGTCTCTCTGTCAGTGAGCAGCGTGACACCTGAGGACACAGCCACATACTACTGTGCAAGAGAcacagtga

>IGHV1S35

CAGGTGCAGCTGCGGGAGTCGGGCCCCAGCCTGGTGAAGCCCTCACAGACCCTCTCCCTCACCTGCACGGTCTCTGGATTCTCATTGAGCAGCTATGCTGTAGGCTGGGTCCGCCAGGCTCCAGGGAAGGCGCTGGAGTGCCTCGGTGGTATAAGCAGTGGTGGAAGCACATACTATAACCCAGCCCTGAAATCACGGCTCAGCATCACCAAGGACAACTCCAAGAGCCAAGTCTCTCTGTCAGTGAGCAGCGTGACACCTGAGGACACGGCCACATACTACTGTGCAAGAGAcacagtga

>IGHV1S36

CAGGTGCAGCTGCAGGAGTCGGGCCCCAGCCTGGTGAAGACCTCACAGACCCTCTCCCTCACCTGCACGGCCTCTGGATTATCATTAACCAGATATGGTATACACTGGGTCCGCCAGGCTCCAGGAAAGGCGCTGGAGTGGCTTGGTGATATAAGCAGTGGTGGAAGCACAGGCTATAACCCAGCCCTGAAATCCAGGCTCAGCATCACCAAGGACAACTCCAAGAGCCAAGTCTCTCTGTCACTGAGCAGCCTGACACCTGAGGACTCAGCCACATACTACTGTGCAAGAGAcacagtga

>IGHV1S37

CAGGTGCAGCTGCAGGAGTCGGGCCCCAGCCTGGTGAAGACCTCACAGACCCTCTCCCTCACCTGCACGGCCTCTGGATTATCATTAACCAGATATGGTATACACTGGGTCCGCCAGGCTCCAGGAAAGGCGCTGGAGTGGCTTGGTGATATAAGCAGTGGTGGAAGCACAGGCTATAACCCAGCCCTGAAATCCCGGCTCAGCATCACCAAGGACAACTCCAAGAGCCAAGTCTCTCTGTCACTGAGCAGCCTGACACCTGAGGACTCAGCCACATACTACTGTGCAAGAGAcacagtga

>IGHV1S38

CAGGTGCAGCTGCAGGAGTCGGGCCCCAGCCTGGTAAAGACCTCACAGACCCTCTCCCTCACCTGCACGGCCTCTGGATTATCATTAACCAGATATGGTATACACTGGGTCCGCCAGGCTCCAGGAAAGGCGCTGGAGTGGCTTGGTGATATAAGCAGTGGTGGAAGCACAGGCTATAACCCAGGCCTGAAATCCCGGCTCAGCATCACCAAGGACAACTCCAAGAGCCAAGTCTCTCTGTCACTGAGCAGCCTGACACCTGAGGACTCAGCCACATACTACTGTGCAAGAGAcacagtga

>IGHV1S39

CAGGTGCAGCTGCGGGAGTCGGGCCCCAGCCTGGTGAAGCCCTCACAGACCCTCTCCCTCACCTGCACGGTCTCTGGATTCTCATTGAGCAGCTATGGTGTAGGCTGGGTCCGCCAGGCTCCAGGGAAGGCGCTGGAGTGTCTTGGTGGTATAAGTAGTGGTGGAAGCACAGGCTATAACCCAGCCCTGAAATCCCGGCTCAGCATCACCAAGGACAACTCCAAGAGCCAAGTCTCTCTGTCACTGAGCAGCGTGACAACTGAGGACACGGCCACATACTACTGTGCGAAGGAcacagtga

>IGHV1S40

CAGGTGCAGCTGCGCGAGTCGGGCCCCAGCCTGGTGAAGCCCTCACAGACCCTCTCCCTCACCTGCACGGTCTCTGGATTCTCATTGAGCAGCTATGCTGTAAGCTGGGTCCGCCAGGCTCCAGGGAAGGCGCTGGAGTGCCTCGGTGGTATAAGCAGTGGTGGAAGCACAGGCTATAACCCAGCCCTGAAATCCCGGCTCAGCATCACCAAGGACAACTCCAAGAGCCAAGTCTCTCTGTCAGTGAGCAGCGTGACACCTGAGGACACGGCCACATACTACTGTGCAAAAGAcacagtga
